# Effect of *Bletilla striata-Litsea cubeba* Toothpaste on Clinical Indicators and Inflammatory Factor Levels in Gingival Crevicular Fluid in Patients with Biofilm-Induced Gingivitis: A Randomised Controlled Trial

**DOI:** 10.3290/j.ohpd.c_2776

**Published:** 2026-09-29

**Authors:** Ruohui Mao, Liang Yu, Xuezhen Gao, Jing Zhao, Yuanna Zheng

**Affiliations:** a Ruohui Mao Dentist, School/Hospital of Stomatology, Zhejiang Chinese Medical University, Hangzhou, Zhejiang, China. Investigation, formal analysis, writing (original draft).; b Liang Yu Dentist, School/Hospital of Stomatology, Zhejiang Chinese Medical University, Hangzhou, Zhejiang, China; Hangzhou TCM Hospital of Zhejiang Chinese Medical University, Hangzhou, Zhejiang, China. Methodology, validation, data curation.; c Xuezhen Gao Dentist, School/Hospital of Stomatology, Zhejiang Chinese Medical University, Hangzhou, Zhejiang, China. Software, visualisation, writing (review and editing).; d Jing Zhao Dentist, School/Hospital of Stomatology, Zhejiang Chinese Medical University, Hangzhou, Zhejiang, China. Writing (review and editing).; e Yuanna Zheng Dentist, School/Hospital of Stomatology, Zhejiang Chinese Medical University, Hangzhou, Zhejiang, China; Ningbo Dental Hospital/Ningbo Oral Health Research Institute, Ningbo, Zhejiang, China. Conceptualisation, writing (review and editing), supervision. All authors reviewed and approved the final manuscript.

**Keywords:** Bletilla striata, gingival crevicular fluid, Litsea cubeba, plaque-induced gingivitis, toothpaste

## Abstract

**Objective:**

To preliminarily evaluate the clinical efficacy of *Bletilla striata-Litsea* cube-batoothpaste in patients with plaque-induced gingivitis and its impact on inflammatory factors in gingival crevicular fluid.

**Methods and Materials:**

In a randomised clinical trial, 80 patients with plaque-induced gingivitis were randomly allocated to receive either *Bletilla striata-Litsea* cubeba toothpaste (experimental group) or negative control toothpaste (control group). Following a 2-week washout period, periodontal indices including the Turesky-modified Quigley–Hein Plaque Index (TmQHI) and Sulcus Bleeding Index (SBI), along with inflammatory cytokines (IL-1β, IL-6, TNF-α) quantified from gingival crevicular fluid (GCF) samples of the right mandibular first molars, were assessed at baseline (0 week), 4, 8, and 12 weeks. Statistical analyses employed repeated-measures analysis of variance (ANOVA) for longitudinal comparisons and independent samples t-tests for inter-group comparisons at identical time points, with statistical significance defined as α = 0.05.

**Results:**

The experimental group exhibited significant TmQHI and SBI reductions at 8 and 12 weeks (*p *< 0.05) versus delayed changes in controls. IL-1β decreased significantly at 8 and 12 weeks with lower levels than controls (*p* < 0.05); IL-6 declined earlier in the experimental group, while TNF-α reductions were comparable between groups.

**Conclusion:**

Compared with control, the novel toothpaste achieved earlier and greater improvements in periodontal indices and specific inflammatory mediator levels in plaque-induced gingivitis.

Plaque-induced gingivitis represents the most prevalent chronic infectious condition among contemporary gingival diseases, exhibiting association with dysbiosis of the periodontal microbiome and dysregulated host immune responses.^11^ Periodontal pathogens such as *Porphyromonas gingivalis* trigger gingival microvasodilation and inflammatory cell infiltration by directly releasing toxins and indirectly inducing host immune responses, increasing the levels of inflammatory factors such as interleukin-1β (IL-1β), interleukin-6 (IL-6), and tumour necrosis factor-α (TNF-α) in gingival crevicular fluid (GCF).^23^ The clinical manifestations are usually dark red gingival colour, bleeding on probing, and round and blunt gingival papilla.

The effectiveness of using toothpaste with brushing for oral cleaning to prevent gingivitis has been supported by research.^12^ As a fundamental oral hygiene intervention, the plaque-inhibitory capacity of toothpaste is contingent upon not merely the mechanical abrasion provided by compounds such as calcium carbonate, but also on its chemical constituents.^1,30
^ For instance, triclosan in toothpaste can reduce dental plaque by interfering with bacterial fatty acid metabolism.^19^ Moreover, many traditional Chinese herbal ingredients, such as clove,^9^
*Andrographis paniculata*
^18^ and gallnut,^6^ have been proven to have significant antibacterial, anti-inflammatory, and anti-sensitive effects, leading to their progressive integration into commercial toothpaste.^4,28,34
^ Nevertheless, current commercially available herbal toothpastes often rely on single plant ingredients, leading to issues such as false advertising, insufficient synergistic effects, and unclear mechanisms of action. For example, Yunnan Baiyao toothpaste has a haemostatic effect, but its formula contains the haemostatic agent tranexamic acid.^24^ These issues highlight the urgent need for scientific optimisation in the formulation of herbal toothpastes and the exploration of new formulations to achieve better antibacterial and anti-inflammatory effects.

*Bletilla striata* is a perennial herb indigenous to traditional Chinese medicine systems and possesses polysaccharides as its primary bioactive constituents. These glucomannan polymers, characterised by β-1,4-glycosidic linkages between mannose and glucose units (molecular weights ranging from 10 to 150 kDa), demonstrate significant antibacterial efficacy, procoagulant activity, and tissue regenerative properties.^14^ Such pharmacological attributes have facilitated their integration into oral care formulations, including lyophilised wafers^17^ and microneedle patches,^25^ enhancing antibacterial performance and promoting oral tissue repair. Concurrently, *Litsea cubeba*, distinguished by its pungent organoleptic properties and warm medicinal nature, contains citral-dominated monoterpenoid compounds within its volatile oil fraction.^8^ Empirical studies validate its marked antibacterial,^16^ anti-inflammatory^33^ and antioxidant activities.^3^
*In vitro* studies indicate that *Litsea cubeba* volatile oil is non-toxic to human gingival fibroblasts and can be added to denture soft lining materials to maintain oral health by inhibiting bacteria.^27^ Given their pharmacological activities and the functional needs of oral care products, combining *Bletilla striata* and *Litsea cubeba* extracts in toothpaste may reduce plaque, stop bleeding, and promote gum healing, assisting in treating plaque-induced gingivitis. Additionally, these extracts can replace thickeners and flavourings in toothpaste, achieving a medicated and auxiliary integration.

Limited research has documented the fundamental physicochemical profiles and therapeutic efficacy of toothpaste incorporating co-extracts of Bletilla striata and Litsea cubeba. This study employs a randomised controlled trial approach to investigate the effects of the toothpaste on clinical periodontal parameters in patients with plaque-induced gingivitis, as well as changes in the concentration of inflammatory mediators in the GCF. The aim is to evaluate the potential anti-plaque and anti-inflammatory effects of the toothpaste, thereby providing scientific evidence for the application of these two herbal extracts in oral care.

## Methods and Materials

### Products for Examination

The *Bletilla striata-Litsea cubeba* toothpaste (Fig 1a, Table 1) was developed by the College of Pharmaceutical Science, Zhejiang Chinese Medical University, and produced in the standardised workshop of Yiwu Aishang Daily Necessities Co. The extract of *Bletilla striata* (provided by Jiangshan Liuheshuo Chinese Medicinal Materials Co., China) is *Bletilla striata polysaccharides* (BSP) (Fig 1b), and the extract of *Litsea cubeba* (provided by Hangzhou Kanglun Chinese Medicine Decoction Pieces Co., China) is *Litsea cubeba* volatile oil (Fig 1c).

**Fig 1a to c Fig1atoc:**
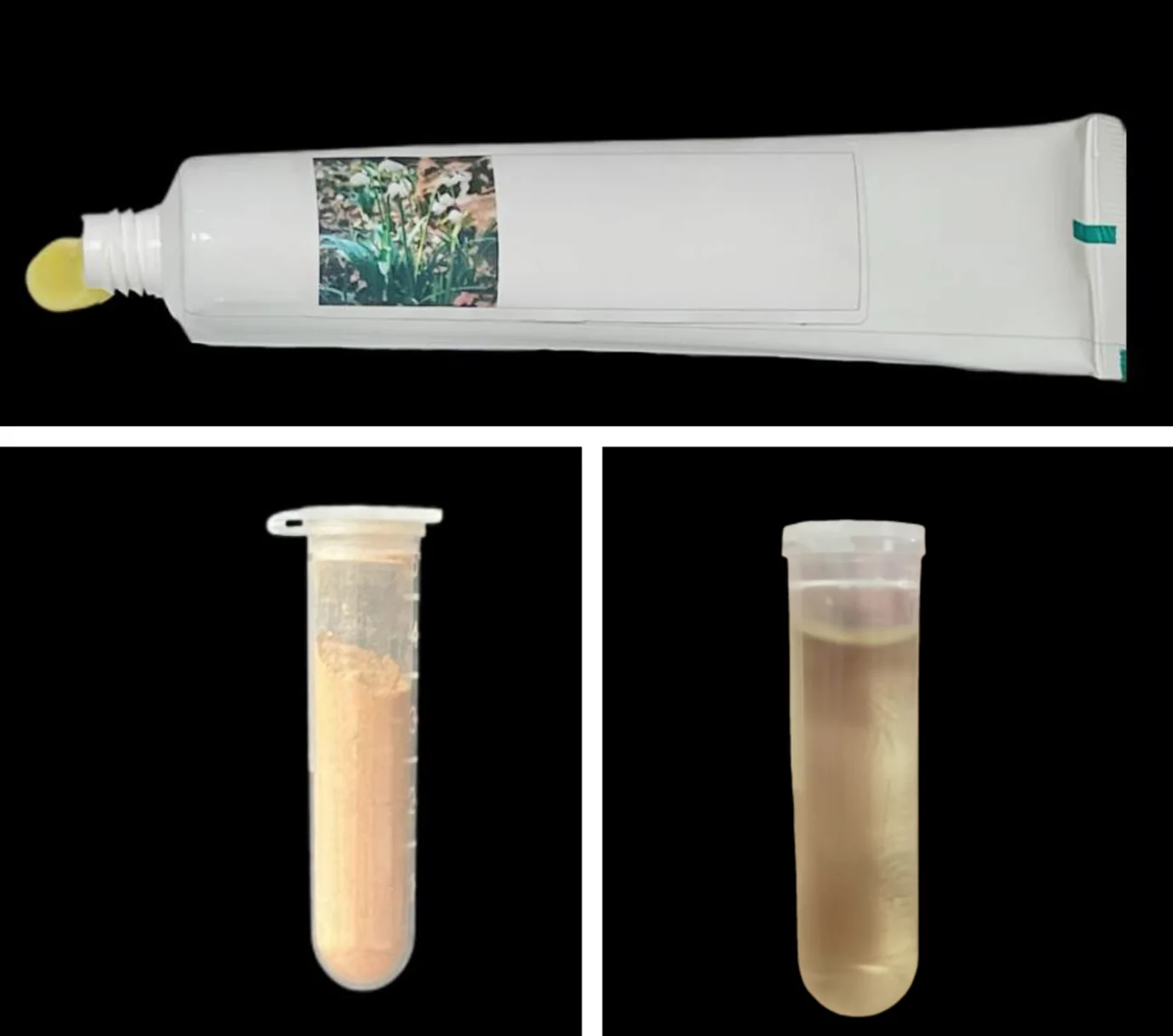
*Bletilla striata-Litsea cubeba* toothpaste: (a) is the appearance of *Bletilla striata-Litsea cubeba* toothpaste; (b) is the polysaccharide extracted from *Bletilla striata*; (c) is the volatile oil of *Litsea cubeba* extract.

**Table 1 Table1:** Composition of *Bletilla striata and Litsea cubeba* toothpaste (per 100 g/tube)

Component	Content (g)	Proportion
Purified water	31.91	31.91%
*Litsea cubeba* β-cyclodextrin inclusion complex	1.17	1.17%
*Bletilla striata* polysaccharide	0.83	0.83%
Sorbitol	48.40	48.40%
Menthol	*	*
Saccharin sodium	*	*
Silicon dioxide 201A	*	*
Silicon dioxide Type H	*	*
Powder K-K_12_	*	*
Tartrazine	0.001	0.001%
Preservative (sodium benzoate)	0.10	0.10%
*represents the patented formulation of the toothpaste, which has not been disclosed during the patent application process.		

The negative control toothpaste, also developed by the College of Pharmaceutical Science, Zhejiang Chinese Medical University, matches the experimental toothpaste in packaging and non-drug trace components, differing only in the absence of Bletilla striata and Litsea cubeba extracts.

### Reagents and Assay Kits

Deionised water, PBS buffer, and human high-sensitivity ELISA kits (IL-6, IL-1β, TNF-α) were sourced from Hangzhou Lianke Biotechnology Co. (Hangzhou, China); plaque disclosing agent was provided by Ci Medical Co. (Osaka, Japan).

### Patients and Study Design

The study was approved by the Ethics Committee of Zhejiang Chinese Medical University (NO.ZCMUHSIRB-2024051407) and registered at the Chinese Clinical Trial Registration Centre (CTR2400086388). All patients provided informed consent.

Patients were recruited from the VIP Department of the Hospital of Stomatology, Zhejiang Chinese Medical University between June 2024 and April 2025. They presented with gingival bleeding and swelling and were clinically diagnosed with plaque-induced gingivitis. All patients signed informed consent before the experiment. Inclusion and exclusion criteria are listed in the table. The Turesky-modified Quigley–Hein Index (TmQHI) was used as the outcome measure. Based on a pre-experiment, the estimated TmQHI was 0.85 ± 0.33 for the experimental group and 1.03 ± 0.27 for the control group.

Using the formula


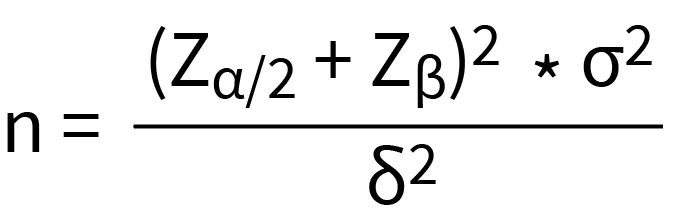
 ,

where σ is the estimated overall standard deviation and β is the difference between the two group means, the sample size was calculated. A two-sided test was used with α = 0.05, β = 0.10, Z_α_ = 1.96, Z_β_ = 1.28, δ = 0.3 (from the pre-experiment), and σ = 0.24. Each group needed 33 subjects. Considering a 20% dropout rate, the sample size was increased.^7^ Eventually, 80 subjects were effectively enrolled (with two dropouts), meeting the requirement.

Patients received oral hygiene education, plaque removal, enamel polishing, and calculus cleaning, if needed.^15^ They learned the modified Bass brushing technique and used a negative control toothpaste for 2 weeks to standardise the baseline. Clinical assessments were conducted at weeks 0 (baseline), 4, 8, and 12, including TmQHI and Sulcus Bleeding Index (SBI) evaluations, GCF collection, and plaque-stained photographic documentation. Patients were randomly assigned to the experimental group or control group by the envelope method. The control group (n = 40) used a negative control toothpaste, while the experimental group (n = 40) used the *Bletilla striata-Litsea cubeba* toothpaste. Both cohorts performed twice-daily brushing for 3 min using the modified Bass technique throughout the 12-week intervention. Toothpaste packaging was identical across groups to maintain participant blinding. This double-masked study restricted allocation awareness exclusively to formulation designers, thereby mitigating assessment subjectivity. All clinical examiners demonstrated inter-rater reliability exceeding the predetermined calibration threshold (Kappa > 0.6), and the study protocol is outlined in Figure 2.

**Fig 2 Fig2:**
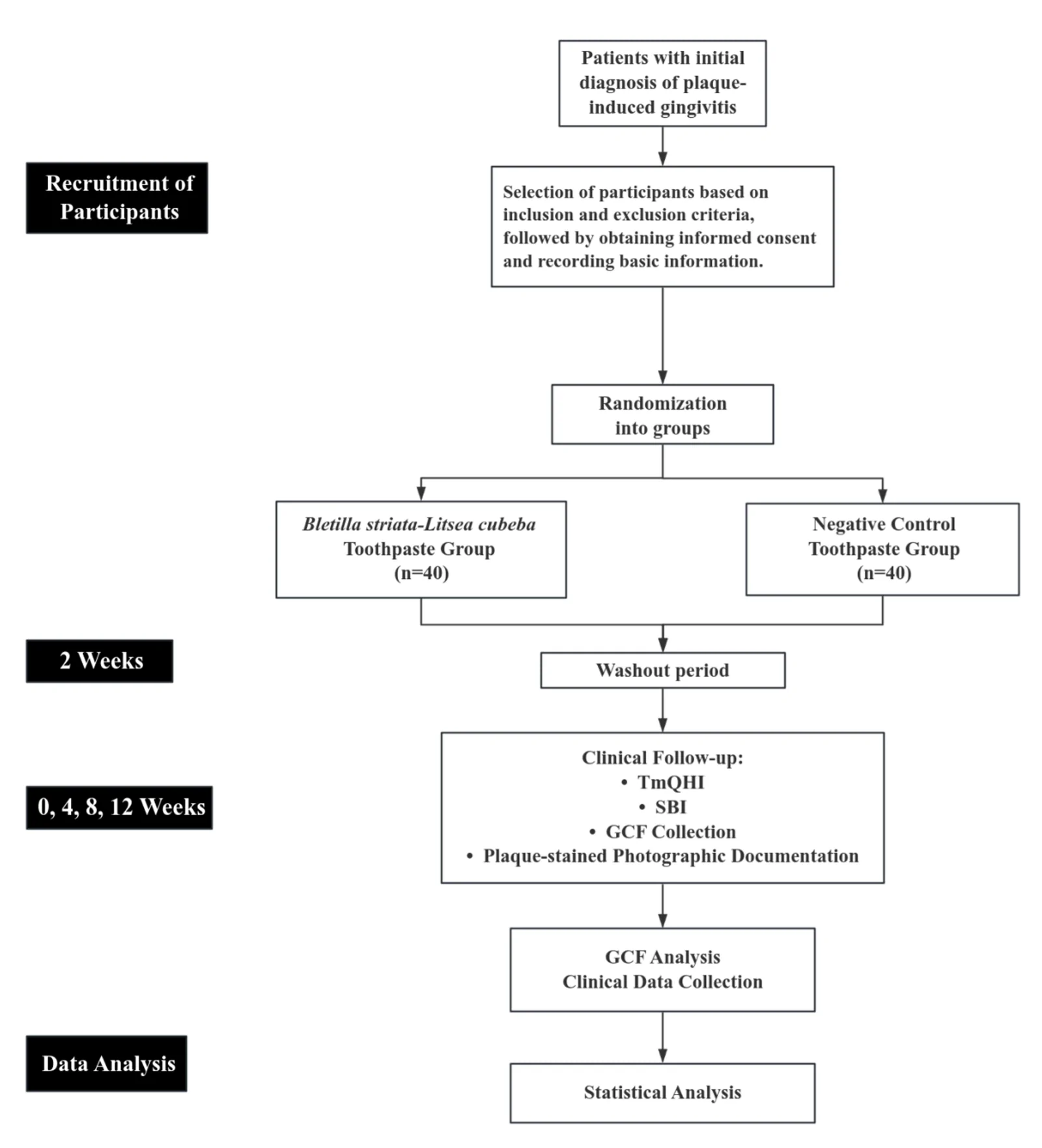
Flow chart of the experiment.

### Efficacy Evaluation

The efficacy evaluation referred to the Chinese-recommended health industry standard *Efficacy Evaluation of Toothpaste (Document No. 25 [2010]*). In each clinical trial using a negative or placebo control, the comparison of final values between the control and experimental groups must show statistical significance. A relative change rate (%) of endpoint value, that is



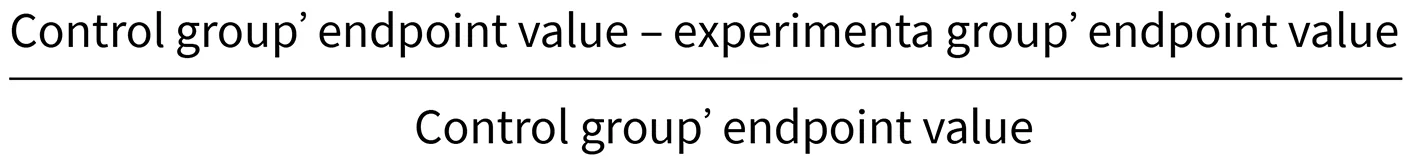



> 12%, and the experimental group must demonstrate a reduction rate of ≥ 15%, indicating the test product’s effectiveness in reducing gingivitis.

### Clinical Assessment

All patients in this study underwent four clinical efficacy evaluations, at 0, 4, 8, and 12 weeks after using the toothpaste from both groups. Each evaluation included measurements of the TmQHI and SBI.

After using a dental plaque disclosing agent, the TmQHI is recorded based on plaque coverage area: 0 indicates no plaque on the tooth surface; 1 indicates scattered plaque spots at the gingival margin; 2 indicates a continuous plaque band (≤ 1mm in width) at the gingival margin; 3 indicates plaque covering > 1 mm but < 1/3 of the tooth surface at the gingival area; 4 indicates plaque covering 1/3 to 2/3 of the tooth surface; 5 indicates plaque covering > 2/3 of the tooth surface.^26,29
^


After probing the gingival sulcus, the bleeding situation was observed to evaluate the BI based on the presence and severity of bleeding. A score of 0 indicates healthy gums with no inflammation or bleeding; 1 indicates inflammatory colour change without bleeding; 2 indicates pinpoint bleeding after probing; 3 indicates bleeding spreading along the gingival margin; 4 indicates bleeding filling and overflowing the sulcus; and 5 indicates spontaneous bleeding.^20^


### Collection of GCF

Four sterile paper points were placed in high-pressure steam-sterilised microcentrifuge tubes and numbered sequentially according to each patient’s name. The target tooth (right lower first molar) was dried with a sterile cotton ball to remove soft debris and saliva. New sterile dry cotton balls were placed on both the buccal and lingual sides of the alveolar ridge to isolate the area and prevent saliva contamination. Using forceps, paper points were inserted approximately 0.5 mm subgingivally at four sites of the target tooth (mesio-buccal, central-buccal, disto-buccal, and lingual), pressed against the tooth neck, and left in place for 30 s. Paper points with blood or saliva contamination were discarded. This process was repeated three times. The three GCF samples in microcentrifuge tubes were used for detecting IL-1β, IL-6, and TNF-α. The paper points were placed in microcentrifuge tubes, sealed, and immediately stored at –80°C. After GCF collection, clinical periodontal examinations were conducted to avoid contamination of the GCF with bleeding from probing.

### Inflammatory Factor Detection

Prior to detection, the samples were restored to room temperature. The absorbent paper points were removed from each microcentrifuge tube, cut into 1 cm segments using sterile scissors, and immersed in 200 μl of PBS buffer for 10 min. The microcentrifuge tubes were then placed in a benchtop high-speed centrifuge for centrifugation (4000 rpm, 10 min, 4°C), and 100 μl of the supernatant was collected for quantification. The levels of IL-1β, IL-6, and TNF-α were measured using ELISA according to the manufacturer’s instructions.

### Statistical Analysis

Statistical analysis was performed using SPSS Statistics 26.0 (IBM, USA). Data within the same group over time were analysed using analysis of variance (ANOVA), while comparisons between two groups at the same time point were conducted using an independent samples t-test. The significance level was defined as α = 0.05.

## RESULTS

The clinical trial enrolled 80 eligible subjects, and no statistically significant differences were observed in baseline characteristics between the two groups (Table 2).

**Table 2 Table2:** Subject information form

Group	Numbers of subjects	Age (years)		Sex		
		Mean ± SD	*p* value	Male	Female	*p* value
Experimental group	40	22.25 ± 5.76	0.174	16	24	0.505
Control group	40	24.25 ± 7.19		19	21	


As shown in Figure 3a, the experimental group showed a progressive and significant reduction in TmQHI from baseline at 8 weeks (*p* < 0.01) and 12 weeks (*p* < 0.001). Within-group comparisons indicated significant differences between 4 and 8 weeks (*p* < 0.05) and 4 and 12 weeks (*p* < 0.001). Although the control group also showed a decreasing trend in TmQHI, the reduction was only significant at 12 weeks (*p* < 0.01), and the change from 4 to 12 weeks (*p* < 0.05) was smaller than in the experimental group. Intergroup comparisons (Fig 3b, Table 3) showed no baseline difference, but the experimental group had significantly lower TmQHI than the control at 8 weeks (*p* < 0.05), with a further increased difference at 12 weeks (*p* < 0.01). Representative plaque-staining follow-up images are shown in Figures 4 and 5. The experimental group demonstrated a greater reduction in TmQHI, declining by 35.97% from baseline at 12 weeks, compared to 20.36% in the control group. The between-group difference of 15.61% was statistically significant, with the experimental group showing a relative improvement of 18.40% at endpoint.

**Fig 3a and b Fig3aandb:**
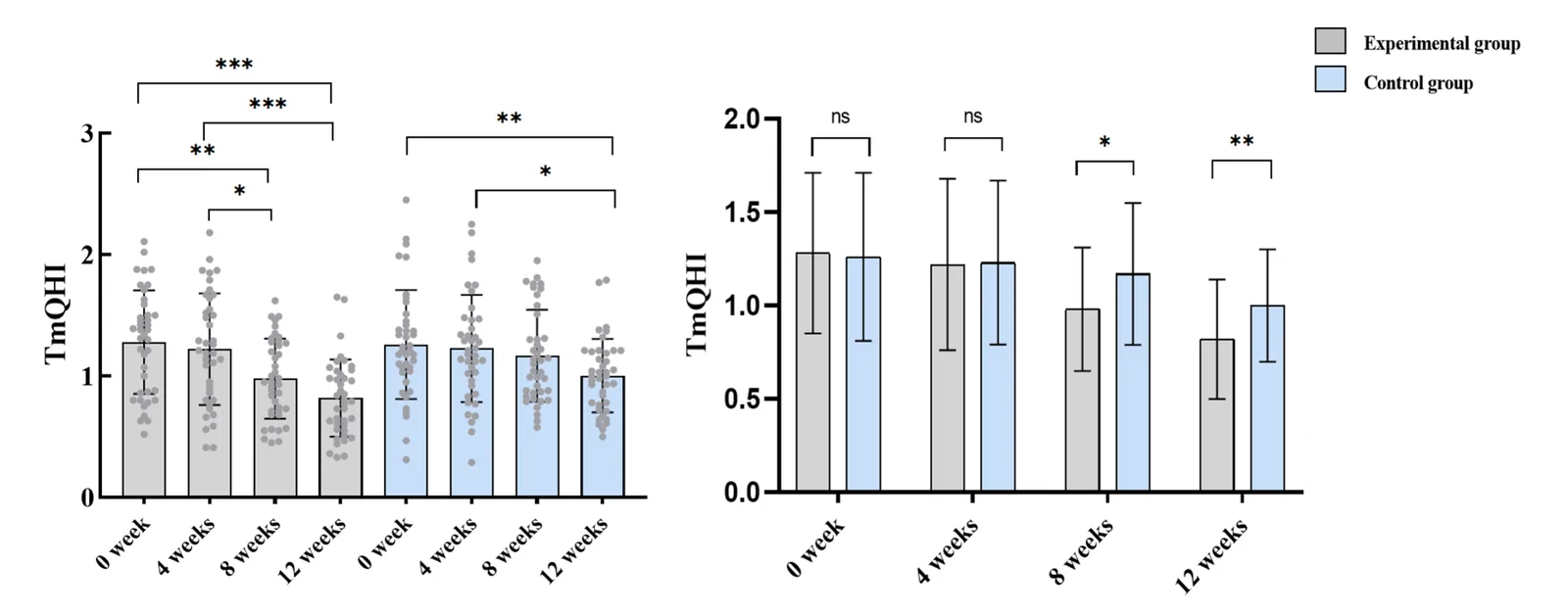
Trends and scores comparison of TmQHI; (a) is changes in TmQHI from baseline over time in two groups; (c) is between-group comparison of TmQHI scores at the same time point ( ‘ns’ indicates no statistical difference, * denotes *p* < 0.05,** denotes *p* < 0.01, and *** denotes *p* < 0.001).

**Table 3 Table3:** Comparison of TmQHI and SBI between two groups at the same time point

Time	TmQHI			SBI		
	Experimental group	Control group	*p* value	Experimental group	Control group	*p* value
0 week	1.28 ± 0.43	1.26 ± 0.45	0.849	0.46 ± 0.42	0.43 ± 0.21	0.667
4 weeks	1.22 ± 0.46	1.23 ± 0.44	0.951	0.36 ± 0.20	0.38 ± 0.21	0.637
8 weeks	0.98 ± 0.33	1.17 ± 0.38	0.020*	0.28 ± 0.15	0.34 ± 0.21	0.172
12 weeks	0.82 ± 0.32	1.00 ± 0.30	0.009**	0.17 ± 0.09	0.35 ± 0.24	0.000***
The independent samples t-test revealed a significant difference between the two groups ( * denotes *p* < 0.05,** denotes *p* < 0.01, and *** denotes *p* < 0.001).						

**Fig 4 Fig4:**
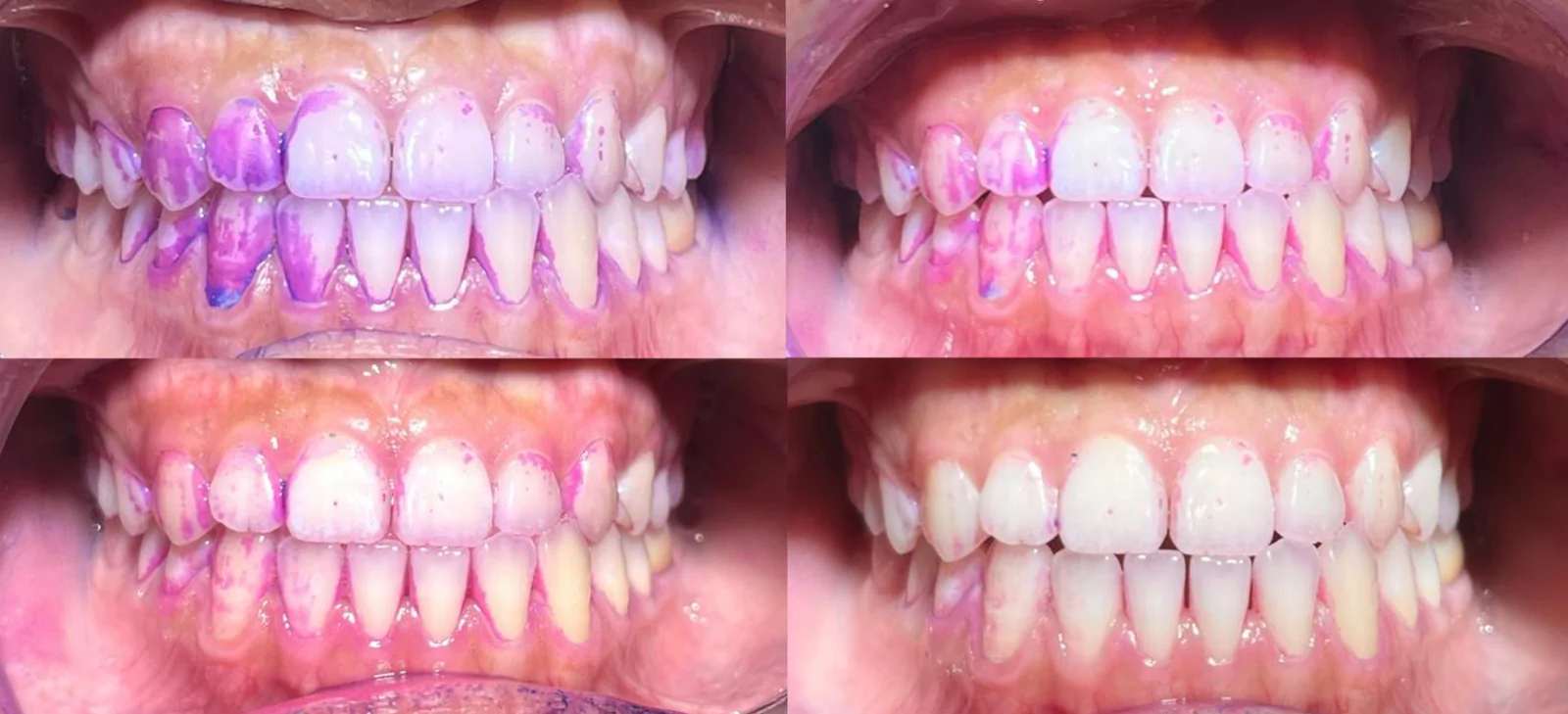
Follow-up photographs of dental plaque staining in the experimental group.

**Fig 5 Fig5:**
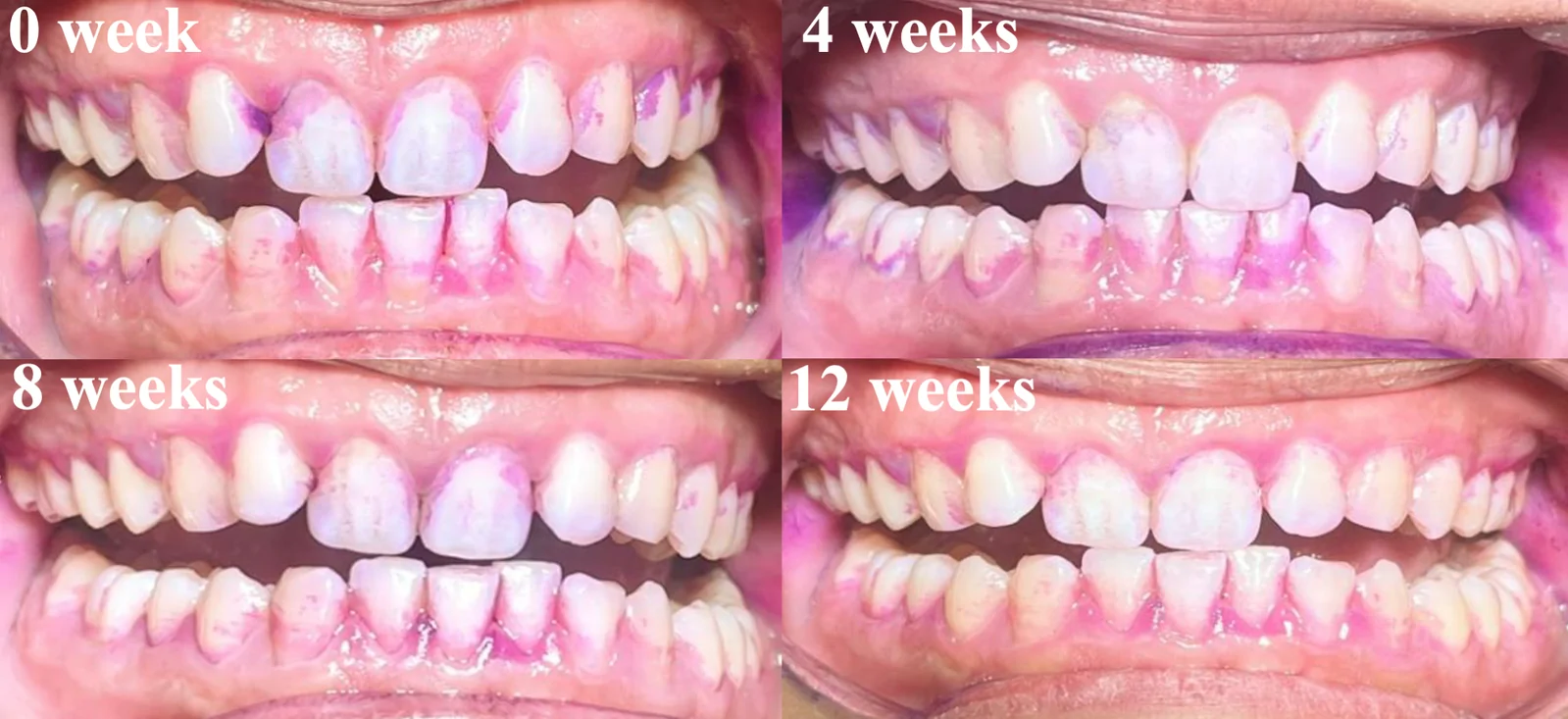
Follow-up photographs of dental plaque staining in the control group.

SBI results showed that the experimental group had significant differences from baseline at 8 weeks (*p* < 0.01), with further increase at 12 weeks (*p* < 0.001) (Fig 6a). Significant differences were also found between 12 weeks and both 4 and 8 weeks (*p* < 0.001, *p* < 0.01). The control group showed no obvious downward trend in SBI throughout the trial, with no significant differences at any time point compared to baseline. Between-group comparison (Fig 6b and Table 3) revealed no significant difference in SBI at baseline. By 12 weeks, the experimental group had a significantly lower SBI than the control group (*p* < 0.001). Compared to baseline, SBI decreased by 63.86% in the experimental group and 19.85% in the control group. The experimental group showed an endpoint reduction rate exceeding 15%, with a between-group difference of 44.01%. Endpoint comparison showed a statistically significant difference between the two groups.

**Fig 6a and b Fig6aandb:**
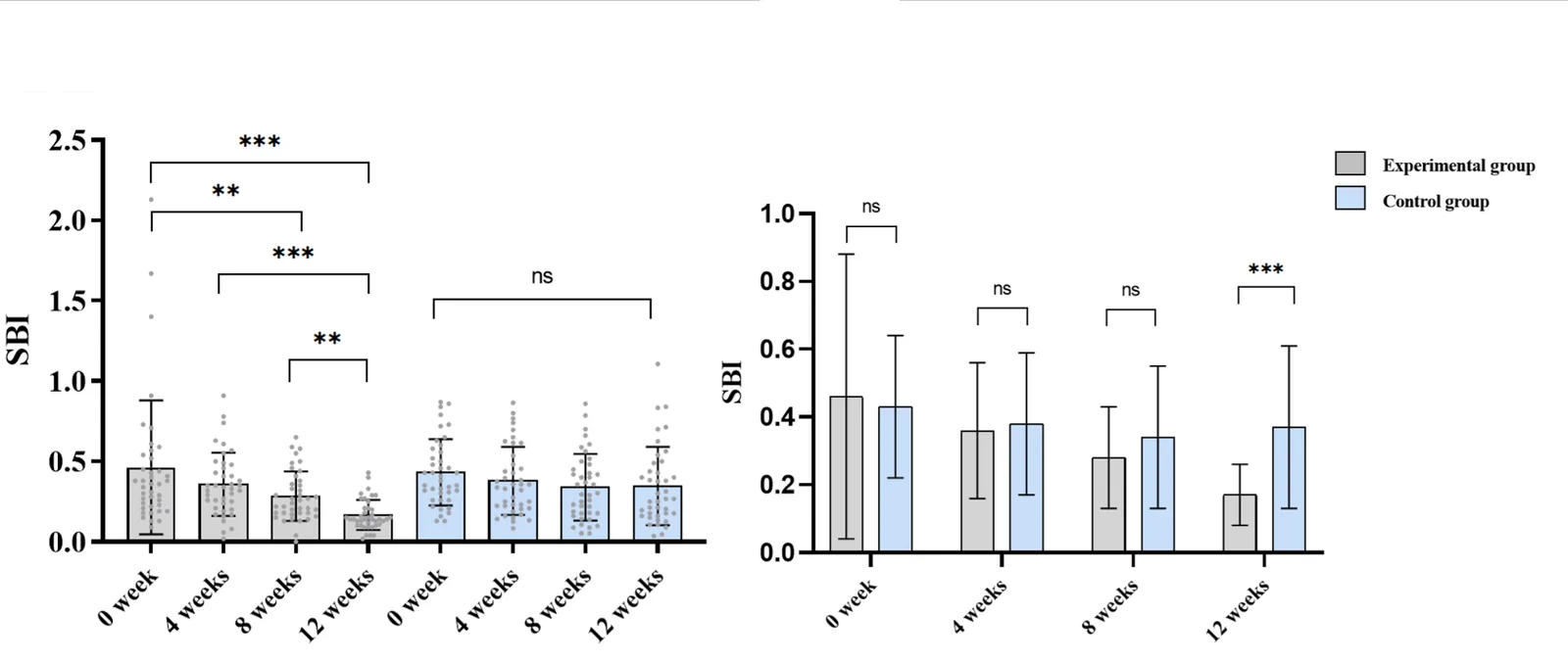
Trends and scores comparison of SBI: (a) is changes in SBI from baseline over time in two groups; (b) is between-group comparison of SBI scores at the same time point ( ‘ns’ indicates no statistical difference; ** denotes *p* < 0.01, and *** denotes *p *< 0.001).

IL-1β results showed a declining trend in both groups. In the experimental group, IL-1β levels in GCF decreased significantly from baseline at 8 weeks (*p* < 0.01) and further at 12 weeks (*p* < 0.001), with a significant difference between 4 and 12 weeks (*p* < 0.001). The control group exhibited a slower decline, showing a significant reduction only at 12 weeks compared to baseline (*p* < 0.01), and a smaller difference between 4 and 12 weeks (*p* < 0.05) (Fig 7a). Intergroup comparisons revealed no baseline difference, but significantly lower IL-1β concentrations in the experimental group at both 8 and 12 weeks (*p* < 0.05) (Fig 7b, Table 4). The reduction rate was 48.82% in the experimental group and 29.89% in the control group. Endpoint comparison showed a statistically significant difference (*p* < 0.05), with a relative change of 29.29%, an experimental group reduction exceeding 15%, and an intergroup reduction difference of 18.93%.

**Fig 7a and b Fig7aandb:**
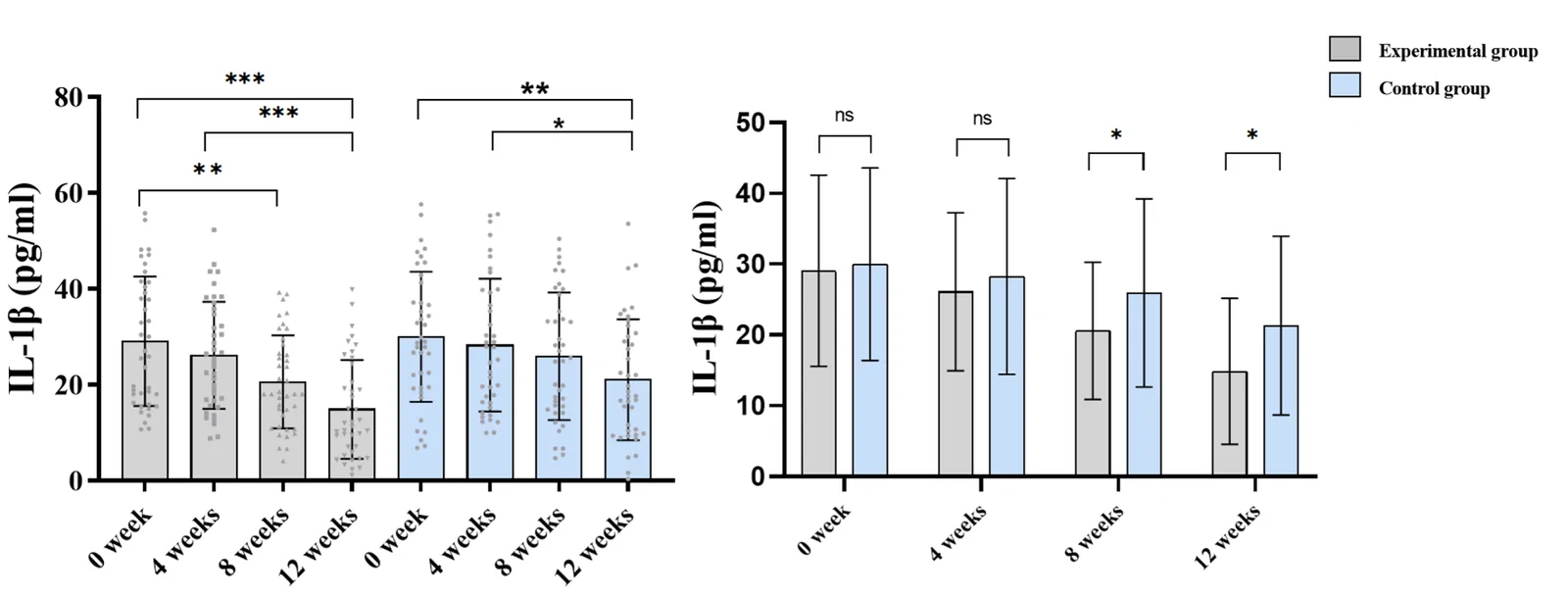
Trends and scores comparison of IL-1β in GCF: (a) is changes in IL-1β levels in GCF from baseline over time between two groups; (b) is between-group comparison of IL-1β levels at the same time point (‘ns’ indicates no statistical difference; * denotes *p* < 0.05, ** denotes *p* < 0.01, and *** denotes *p* < 0.001).

**Table 4 Table4:** Comparison of IL-1β, IL-6, and TNF-α levels between two groups at the same time point

Time	IL-1β (pg/ml)		IL-6 (pg/ml)			TNF-α (pg/ml)			
	Experimental group	Control group	*p* value	Experimental group	Control group	*p* value	Experimental group	Control group	*p* value
0 week	29.06 ± 13.50	30.00 ± 13.59	0.757	9.37 ± 4.86	9.78 ± 4.52	0.697	15.48 ± 6.87	17.16 ± 9.19	0.357
4 weeks	26.12 ± 11.17	28.27 ± 13.85	0.447	8.51 ± 3.82	9.28 ± 4.68	0.421	13.64 ± 6.64	15.32 ± 9.57	0.364
8 weeks	20.58 ± 9.69	25.93 ± 13.30	0.043*	7.44 ± 3.92	8.73 ± 5.21	0.213	10.63 ± 5.72	12.76 ± 8.44	0.191
12 weeks	14.87 ± 10.31	21.30 ± 12.63	0.019*	6.07 ± 4.19	7.58 ± 4.56	0.127	8.35 ± 6.02	10.74 ± 7.82	0.130
The independent samples t-test revealed a significant difference between the two groups ( * denotes *p* < 0.05).									

Both groups exhibited declines in IL-6 levels (Fig 8a). The experimental group demonstrated significant decreases at 8 weeks (*p* < 0.05) and 12 weeks (*p* < 0.01) relative to baseline, with a significant change between 4 and 12 weeks (*p* < 0.05). The control group also showed a reduction, though less pronounced, reaching significance only at 12 weeks compared to baseline (*p* < 0.05). Intergroup comparisons (Fig 8b, Table 4) revealed no significant differences at any time point, despite a downward trend in *p* values over time. The experimental group achieved a 35.18% reduction in IL-6 levels, compared to 22.46% in the control group. Despite a 19.91% relative intergroup difference at the endpoint, a greater than 15% decline rate in the experimental group, and a 12.72% difference in overall reduction, the endpoint comparison between groups was not statistically significant.

**Fig 8a and b Fig8aandb:**
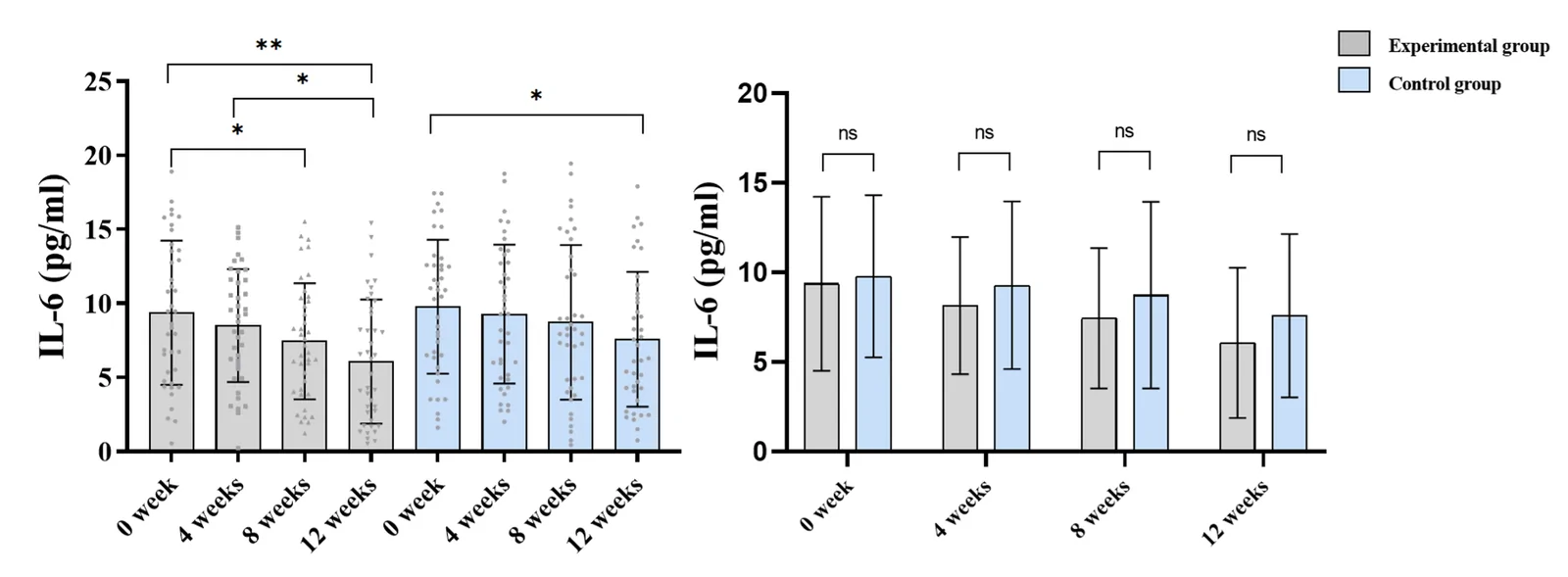
Trends and levels comparison of IL-6 in GCF: (a) is changes in IL-6 levels in GCF from baseline over time between two groups; (b) is between-group comparison of IL-6 levels at the same time point ( ‘ns’ indicates no statistical difference; * denotes *p* < 0.05, ** denotes *p* < 0.01).

TNF-α levels in GCF declined in both groups (Fig 9a). The experimental group demonstrated significant reductions at 8 weeks (*p* < 0.01) and 12 weeks (*p* < 0.01) vs baseline, with differences between 4 weeks vs 8 and 12 weeks (*p* < 0.05). The control group showed similar trends: TNF-α decreased significantly at 8 weeks (*p* < 0.05) and 12 weeks (*p* < 0.01) vs baseline, with a difference between 12 weeks and 4 weeks (*p* < 0.05). Intergroup comparisons (Fig 9b, Table 4) revealed no significant differences at any time point, but *p* values progressively decreased over time. The results demonstrate that the experimental group achieved a 46.06% reduction in TNF-α levels, while the control group showed a 37.46% decline. Although TNF-α endpoints exhibited a 22.21% relative change, the experimental group surpassed the 15% reduction threshold, and the between-group difference in decline rates reached 8.60%, the endpoint comparison revealed no statistically significant differences between the groups.

**Fig 9a and b Fig9aandb:**
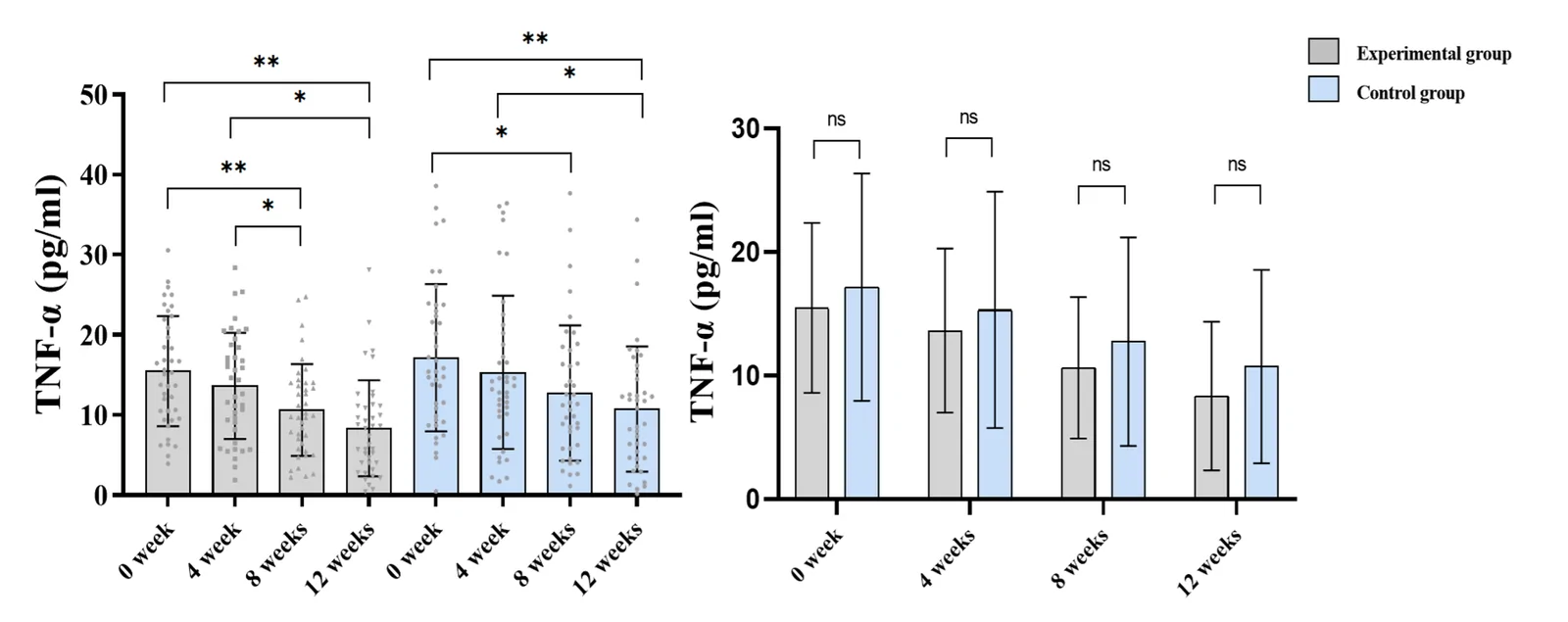
Trends and levels comparison of TNF-α in GCF: (a) is changes in TNF-α levels in GCF from baseline over time between two groups; (b) is between-group comparison of TNF-α levels at the same time point ( ‘ns’ indicates no statistical difference; * denotes p < 0.05, ** denotes *p* < 0.01).

## Discussion

This prospective clinical study compared the efficacy of *Bletilla striata-Litsea cubeba* toothpaste versus a negative control toothpaste. The null hypothesis was partially rejected, with statistically significant differences between the two toothpastes in reducing TmQHI, SBI, and IL-1β levels.

This clinical investigation compared the effects of *Bletilla striata-Litsea cubeba* toothpaste versus negative control toothpaste, demonstrating the experimental group’s significant efficacy in reducing both TmQHI and SBI scores. Both groups exhibited reductions in TmQHI, potentially associated with the implementation of the modified Bass technique for three-minute brushing sessions. Research has demonstrated that this method increases plaque removal efficacy by 55% when compared to 30-s brushing.^5^ The experimental group’s enhanced efficacy likely stemmed from the synergistic antibacterial effects of BSP and *Litsea cubeba* volatile oils. BSP disrupt bacterial cell walls and membranes, causing ion leakage and metabolic dysfunction,^10^ while *Litsea cubeba* oils inhibit pathogens like *S. aureus* and *E. coli* through membrane destabilisation.^13,22
^ Only the experimental group demonstrated efficacy in reducing gingival bleeding. This may be related to BSP’s haemostatic properties, which promote vascular endothelial growth factor (VEGF) expression,^31^ accelerate clotting via extrinsic and intrinsic pathways, and modulate thromboxane-prostacyclin balance.^36^


Experimental results indicated that *Bletilla striata-Litsea cubeba* toothpaste demonstrated efficacy in reducing IL-1β levels, while revealing no significant effects on IL-6 and TNF-α modulation. Both experimental and control cohorts exhibited reductions in inflammatory cytokine levels (IL-1β, IL-6, TNF-α) within GCF, with the experimental group demonstrating superior reduction efficacy and accelerated onset speed. This phenomenon may be attributed to the anti-inflammatory properties of BSP and *Litsea cubeba* essential volatile oil. Experimental evidence from murine models confirms that BSP mitigates high glucose-induced reactive oxygen species (ROS) elevation in bone marrow-derived macrophages (BMDMs), concurrently suppressing NLRP3 inflammasome overactivation and IL-1β hypersecretion.^37^ Animal experiments conducted by Gong Zihan et al have also demonstrated that BSP can inhibit the overexpression of inflammatory cytokines IL-1β, IL-6, and TNF-α by modulating the expression levels of ERK, JNK, and p38 MAPK, which are subgroups of MAPK.^38^
*Litsea cubeba* volatile oil potentially inhibits IL-6 and TNF-α through JAK2/STAT3 and NF-κB signalling pathways, while attenuating IL-1β and IL-6 levels via NLRP3 inflammasome inhibition.^32,35
^ Although both compounds target the NLRP3 inflammasome, potential signalling pathway convergence and synergistic anti-inflammatory mechanisms remain unexplored, warranting further investigation.

Only IL-1β levels exhibited statistically significant intergroup divergence, potentially attributable to differential sensitivity among inflammatory mediators. Research indicates that 70% of investigators regard IL-1β as the most responsive biomarker in gingivitis contexts, with its concentrations in GCF substantially exceeding those of other cytokines (e.g.TNF-α, IL-6, and IL-8), thereby establishing it as the most representative diagnostic marker of gingivitis severity.^2^ Differences in the detection capabilities of ELISA kits and GCF volume may also lead to varying result sensitivity.^21^ The observed attenuation of IL-6 and TNF-α in controls may be linked to standardised brushing protocols that mechanically reduce plaque accumulation and consequent local inflammation. Although terminal endpoint values for IL-6 and TNF-α failed to attain statistical significance between cohorts, this may reflect insufficient observational duration. Notwithstanding the nonsignificant *p* values, both cytokines demonstrated progressive downward trajectories (Table 4), suggesting that prolonged intervention would potentially amplify intergroup differences.

A limitation of this study was the selection of mostly young and middle-aged patients, resulting in a homogeneous sample that restricts the generalisability of the findings. Future research should include a more diverse group of participants with varying oral health conditions, ages, and genders to enhance the universality and reliability of the results. Additionally, the periodontal microenvironment is a dynamic and slowly remodelling process, and plaque-induced gingivitis is a chronic condition that develops and recurs over extended periods. A 12-week observation might be too brief to fully assess the toothpaste’s long-term efficacy. Future studies should consider longer observation periods to evaluate the toothpaste’s sustained effects and post-discontinuation recurrence rates, offering a more comprehensive effectiveness assessment. Lastly, the absence of positive and single-herb control groups in this experiment made it impossible to compare the experimental toothpaste with existing products or assess the individual effects of *Bletilla striata* and *Litsea cubeba* components. Future research should incorporate positive and single-ingredient control groups to strengthen the clinical evaluation evidence.

## Conclusion

During the 12-week clinical trial period, *Bletilla striata-Litsea cubeba* toothpaste demonstrated statistically significant efficacy in reducing TmQHI, SBI, and IL-1β levels, but showed no significant effects on IL-6 or TNF-α level modulation. Future studies should enhance the diversity of the study population and extend the intervention duration to comprehensively evaluate the toothpaste’s clinical effects.

### Acknowledgements

The author gratefully acknowledges all the doctors for their participation in this study.

#### Data availability statement

The data that support the findings of this study are available from the corresponding author upon reasonable request.

#### Funding statement

This work was supported by Zhejiang Province Hangzhou Qianjiang Distinguished Expert Support Project, Grant Number: 10261.

#### Conflict of interest disclosure

The authors declare no conflicts of interest.

#### Ethics approval statement

All study procedures involving human participants were performed in accordance with the ethical standards of the Institutional Research Ethics Committee of Hospital of Stomatology, Zhejiang Chinese Medical University (ZCMUHISRB-2024051407).

#### Patient consent statement

All experiments involved patient consent, and informed consent was obtained.

## References
